# HIV-1 assembly in macrophages

**DOI:** 10.1186/1742-4690-7-29

**Published:** 2010-04-07

**Authors:** Philippe Benaroch, Elisabeth Billard, Raphaël Gaudin, Michael Schindler, Mabel Jouve

**Affiliations:** 1 Institut Curie, Centre de Recherche, Paris, F-75248 France; INSERM U932, Paris, F-75248 France; 2 Heinrich-Pette-Institut, Martinistrasse 52, 20251 Hamburg, Germany; 3 Institut Jacques Monod. 75205 PARIS cedex 13, France

## Abstract

The molecular mechanisms involved in the assembly of newly synthesized Human Immunodeficiency Virus (HIV) particles are poorly understood. Most of the work on HIV-1 assembly has been performed in T cells in which viral particle budding and assembly take place at the plasma membrane. In contrast, few studies have been performed on macrophages, the other major target of HIV-1. Infected macrophages represent a viral reservoir and probably play a key role in HIV-1 physiopathology. Indeed macrophages retain infectious particles for long periods of time, keeping them protected from anti-viral immune response or drug treatments. Here, we present an overview of what is known about HIV-1 assembly in macrophages as compared to T lymphocytes or cell lines.

Early electron microscopy studies suggested that viral assembly takes place at the limiting membrane of an intracellular compartment in macrophages and not at the plasma membrane as in T cells. This was first considered as a late endosomal compartment in which viral budding seems to be similar to the process of vesicle release into multi-vesicular bodies. This view was notably supported by a large body of evidence involving the ESCRT (Endosomal Sorting Complex Required for Transport) machinery in HIV-1 budding, the observation of viral budding profiles in such compartments by immuno-electron microscopy, and the presence of late endosomal markers associated with macrophage-derived virions. However, this model needs to be revisited as recent data indicate that the viral compartment has a neutral pH and can be connected to the plasma membrane via very thin micro-channels. To date, the exact nature and biogenesis of the HIV assembly compartment in macrophages remains elusive. Many cellular proteins potentially involved in the late phases of HIV-1 cycle have been identified; and, recently, the list has grown rapidly with the publication of four independent genome-wide screens. However, their respective roles in infected cells and especially in macrophages remain to be characterized. In summary, the complete process of HIV-1 assembly is still poorly understood and will undoubtedly benefit from the ongoing explosion of new imaging techniques allowing better time-lapse and quantitative studies.

## Review

### Role of monocytes/macrophages in HIV-1 physiopathology

Rapidly after the discovery of HIV-1, it was established that HIV-1 has two major targets in vivo; T lymphocytes, which have been extensively studied, and macrophages. While the viral replication cycle is usually rapid and cytopathic in T cells, infected macrophages survive for months *in vitro *and *in vivo*, and accumulate large vacuoles containing infectious viral particles [1-3]. HIV-1 enters the Central Nervous System (CNS) soon after peripheral infection of circulating T cells and monocytes and probably penetrates the CNS at various times during infection, see review [4]. Immunohistochemistry and *in situ *hybridization studies have demonstrated that, in the CNS, perivascular macrophages and microglia are the most productively HIV-infected cells and are likely to mediate CNS dysfunctions observed in individuals infected with HIV-1 [4]. Intracellular location has long been considered to provide a privileged niche, protecting the virus from the immune system as well as from the action of antiviral drugs. Thus, HIV-1 can persist in a protected brain reservoir made of infected monocytes/macrophages despite anti-retroviral therapy. Therefore upon arrest of highly active antiretroviral therapy, macrophages but also blood monocytes [5] may contribute to the spread of HIV-1 and the rapid reconstitution of high viral loads.

Macrophages differentiate from monocytes and represent a very diverse population of phagocytes, present in many tissues and involved in various functions (from bone remodeling to muscle regeneration, see review [6]) acting in both innate and adaptive immunity. Their first function is to phagocytose cellular debris and pathogens either as stationary or mobile cells. Therefore, they possess a very active endo-lysosomal system, the activity and rapidity of which may have been underestimated. Looking at the ultra-structural level at human macrophages, one is struck by the richness of the endo-lysosomal network and the paucity of intermediate compartments suggesting that internalized materials are very rapidly targeted to lysosomes [7].

#### Scope of the present review

Despite the importance of macrophages for the physiopathology of AIDS, and the initial interest after their identification as the second main target of the virus *in vivo*, very little is known about the HIV-1 cycle in macrophages. Most studies have been performed in non-macrophage cell lines. and it is unclear whether such results hold true in macrophages. Here, we will review the HIV assembly process within infected primary macrophages, *i.e. most commonly, monocyte-derived macrophages*.

### Current view(s) of HIV-1 assembly

#### Coordinating viral assembly

**Figure 1:**
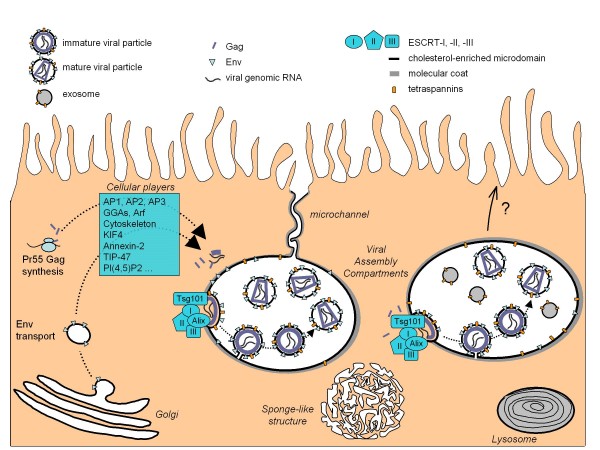
**A current view of HIV assembly in macrophages**. The viral genomic RNA transcribed in the nucleus is exported to the cytoplasm. The transmembrane envelope (Env) protein is produced in the endoplasmic reticulum and transits through the Golgi apparatus while Gag is synthesized on free cytosolic ribosomes. Both Env and the Gag precursors are targeted to the assembly site through unidentified pathways. The sites of Gag/Env interaction, Gag multimerization and binding to viral genomic RNA remain elusive as well. The main cellular factors suspected to play a role in these trafficking events are indicated; nevertheless most of the time their roles have still to be established in macrophages. The assembly process requires the hijacking of the cellular ESCRT machinery and occurs on cholesterol- and tetraspanin-enriched membrane microdomains. The assembly compartment can be connected at least transiently to the plasma membrane through thin microchannels that do not allow virion passage. The limiting membrane of the viral assembly compartment as well as the microchannels often exhibit thick molecular coats of which the composition remains obscure. See text for details.

In this section, we focus on the late events of viral replication in macrophages. Currently, it remains unclear how the various components of the viral particle are targeted to the assembly compartment of which the exact nature and localization remain elusive (see Figure 1 for a summary). Early studies showed that infected macrophages tend to accumulate intracellular vacuoles that contain numerous viral particles [1,2,8]. Since budding events have been observed at the limiting membranes of these vacuoles, [9,10], they are generally considered as the site of HIV-1 assembly in macrophages. We will refer to these vacuoles as the viral assembly compartment in the present review.

The trafficking of viral components to the assembly site as well as their subsequent assembly and release in the form of an infectious particle are coordinated and regulated through interactions between viral structural proteins and cellular factors. The product of the *gag *gene has long been recognized as the main conductor of HIV-1 assembly since its expression alone gives rise to virus-like particles having the same spherical shell structure as immature viral particles [11,12]. The current view of HIV-1 assembly in T cells has been recently reviewed [13,14], and we will only give here a brief overview of the process.

Gag is composed of three polypeptides-- the matrix, the capsid, the nucleocapsid; and three smaller peptides that function together to coordinate membrane binding and Gag-Gag lattice interactions in immature virions [15]. One of the three peptides is called p6 or the "late domain" because it is required for virus budding and release [16]. The Gag precursor is synthesized in the cytosol and co-translationally myristoylated at its N-terminus, which is required for stable membrane association. It is then targeted to the cytoplasmic leaflet of membranes through mechanisms that are not fully understood. There, Gag multimerizes into microdomains, which in turn stabilize its membrane association [17].

Gag can be found in the cytosol as small oligomers detected by immuno-EM [18], but it is not known whether Gag oligomerization is a prerequisite for the spherical Gag lattice formation. Similarly, it remains unclear whether the transport of the precursor relies on free cytoplasmic diffusion or if it requires trafficking along the cytoskeleton. It has also been suggested that RNA binding to Gag could play a role in the assembly process by providing a scaffold to stabilize intermolecular Gag interactions [19,20]. Where and when the interaction between Gag and viral RNA occurs is still debated, but the trafficking of genomic RNA may influence Gag cytosolic fate [21-24]. Of note, the majority of data concerning intracellular Gag trafficking was obtained from immortalized cell lines and does not necessarily reflect the situation in infected primary macrophages.

#### Host factors involved in assembly

Among the numerous cellular factors reported to be involved in HIV-1 assembly and budding, the ESCRT cellular machinery (Endosomal Sorting Complex Required for Transport) is recruited by the p6 domain and plays a key role in the formation and release of new particles. This complex has drawn a lot of attention, and much progress has been made in the last few years in understanding its way of functioning in three important processes: formation of intraluminal vesicles in multi-vesicular bodies (MVBs), HIV-1 budding and fission from membranes, and more recently in fission of the midbody during cytokinesis. The three processes have in common the need for severing a thin membrane to allow vesicles, nascent viral particles, or cells to be released. Since this large body of work has not been reproduced in macrophages and because the mechanisms involved have been thoroughly reviewed [13,15,25], they will not be discussed here.

Additionally, Vpu, one of the accessory proteins of HIV-1, also plays a crucial role in the terminal step of particle release (see [13]). Indeed, Vpu has been recently shown to counteract the activity of a restriction factor named tetherin/BST-2/CD317 [26-30]. In the absence of Vpu, viral particles bud from the plasma membrane of T cells but cannot detach due to the presence of tetherin. The action of Vpu in T cells may rely on the down-regulation of BST-2 at the cell surface through both relocalization and degradation of this factor [31-33]. The molecular mechanism involved in this tetherin-mediated retention remains unknown as well as the exact role of Vpu in different cell types, especially in macrophages [32].

##### Other cellular players

In addition to the ESCRT machinery many cellular proteins are thought to be recruited or affected for efficient viral assembly and release [15,34]. Only a few of those factors have been characterized in macrophages. One of them is a cholesterol transporter named ABCA1, which when bound to Nef could result in the impairment of cholesterol efflux in infected macrophages [35]. This may be related to the requirement of cholesterol in the viral envelope for better infectivity. Another factor reported to be essential for both productive infection of macrophages and the infectivity of released virions is Annexin2 which binds to Gag at the limiting membrane of the viral assembly compartment [36]. Annexin 2 seems to be involved in many functions including membrane trafficking and endosome formation, and its intracellular distribution depends on cholesterol [37]. Since Annexin2 is not expressed by lymphocytes, its expression in macrophages may contribute to the particular localization of their viral assembly site.

Studies performed with cells other than macrophages have revealed many proteins involved in the trafficking of Gag or Env towards the assembly site or its regulation, such as Clathrin adaptors AP-1, AP-2 and AP-3 [38-44], clathrin-binding factors GGAs and their regulator Arf [45] and TIP47, which could simultaneously bind to Env and Gag [46]. The microtubule network could play a role via the inducible host factor SOCS1 in the intracellular trafficking of Gag [47-49], as well as the kinesin KIF4 which binds to Gag and is required for viral assembly [50,51]. Moreover, a thorough proteomic analysis of purified virions produced by HIV-1-infected macrophages showed the presence of numerous of these host proteins [52].

The above list of cellular proteins involved is far from exhaustive. Recently, siRNA-based genome-wide screens by 4 independent teams have identified cellular proteins potentially involved at various stages of the viral cycle [53-56]. These studies have produced large numbers of candidates of which very few overlap. This may reflect, in part, differences in the experimental set up used for each of these screens which used different HIV-1 isolates and cell lines (HEK293T or HeLa cells and Jurkat cells; see meta-analysis [57] and comment [58]). While many proteins have been proposed to play roles in the HIV-1 assembly process, their respective contributions and the temporal order of the events are far from established.

### Approaching HIV-1 assembly in primary macrophages

#### Technical limitations

**Figure 2:**
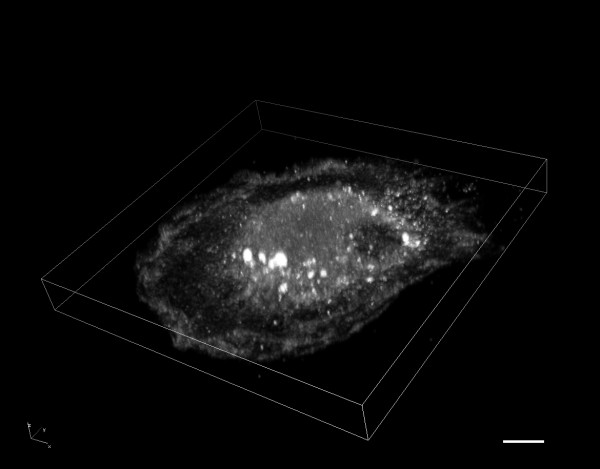
**Immunofluorescent staining of Gag in a HIV-1-infected macrophage**. Monocyte-derived-macrophages were infected with HIV-1 NLAD8 pseudotyped with VSV-G. At day 7 post-infection, cells were fixed, permeabilized and stained with a rabbit antiserum anti-HIV-1 p17 (AIDS Research and Reference Reagent Program, Division of AIDS, NIAID, NIH, from Dr. Paul Spearman) revealed by goat anti-rabbit antibodies conjugated to Alexa Fluor 488. A three dimensional reconstruction built from an 8 μm thick section (0.5 μm between planes) is presented. It has been generated using the Nikon A1R Confocal laser microscope system. The macrophages often appear with this typical shape in "sunny side up egg" where the nucleus is a small part of the "yolk". The Gag staining appears rich and complex; there is a diffuse cytosolic staining, some structures with intense staining located in the "yolk" which may correspond to the viral assembly compartments, and very small dots scattered everywhere which could correspond to free virions or Gag multimers (the microscope resolution is not good enough to estimate their precise size). Scale bar, 5 μm.

Many studies have been based on immuno-fluorescent staining of viral proteins such as Gag in infected macrophages. In fact, Gag has multiple localizations in infected cells (see an example in Figure 2, typical of day 7 post-infection). Gag goes from a diffuse cytosolic pattern to small dots in the periphery to large intracellular compartments. Moreover, the Gag staining pattern evolves with time post-infection. Two additional reasons render the interpretation of these staining even more complex:

i) The poor resolution of the epifluorescent microscopy technique does not allow one to distinguish mature or nascent viral particles from Gag aggregates. Note that the diameter of an immature viral particle is in the range of 100 to 200 nm (mean 129 nm) [59], which is below the resolution of epifluorescent microscopes.

ii) It is impossible to distinguish incoming virions, which may fuse or be internalized, from nascent viral particles eventually being secreted. Similarly, there is no way to know whether dots observed by immunofluorescence represent infectious or non-infectious particles. Finally, we do not know if all the synthesized Gag precursor has a homogeneous behavior or if several populations of Gag precursor exist with distinct fate and function. This idea is supported by Gousset et al. showing that only part of Gag was redistributed in infected macrophages towards the synapse formed with non-infected T cells [60].

Some of these problems can be, in theory, circumvented by ultrastructural approaches. So far, only Immuno-electron microscopy (Immuno-EM) allows one to distinguish viral particles, from viral buds, and from non-assembled Gag. However, this technique remains tedious, difficult to master, and only works with very few antibodies on fixed samples.

#### How ultrastructural studies have shaped our representation of HIV-1 assembly in macrophages

EM studies have greatly influenced our view of the viral cycle in macrophages. Early work revealed the existence of large intracellular vacuoles in which viral particles tend to accumulate. Raposo et al. showed by immuno-EM that these vacuoles contained not only virions, but also endosomal markers such as MHC II and CD63. Based on EM profiles they also proposed that viral budding takes place at the limiting membrane of the compartments, and that fusion of these compartments can occur at the plasma membrane leading to the release of their contents; HIV-1 particles and exosomes [9]. Pelchen-Matthews et al. confirmed these results and provided additional biochemical evidence that viral particles originate from late endocytic compartments and carry markers from these compartments [10,61].

**Figure 3:**
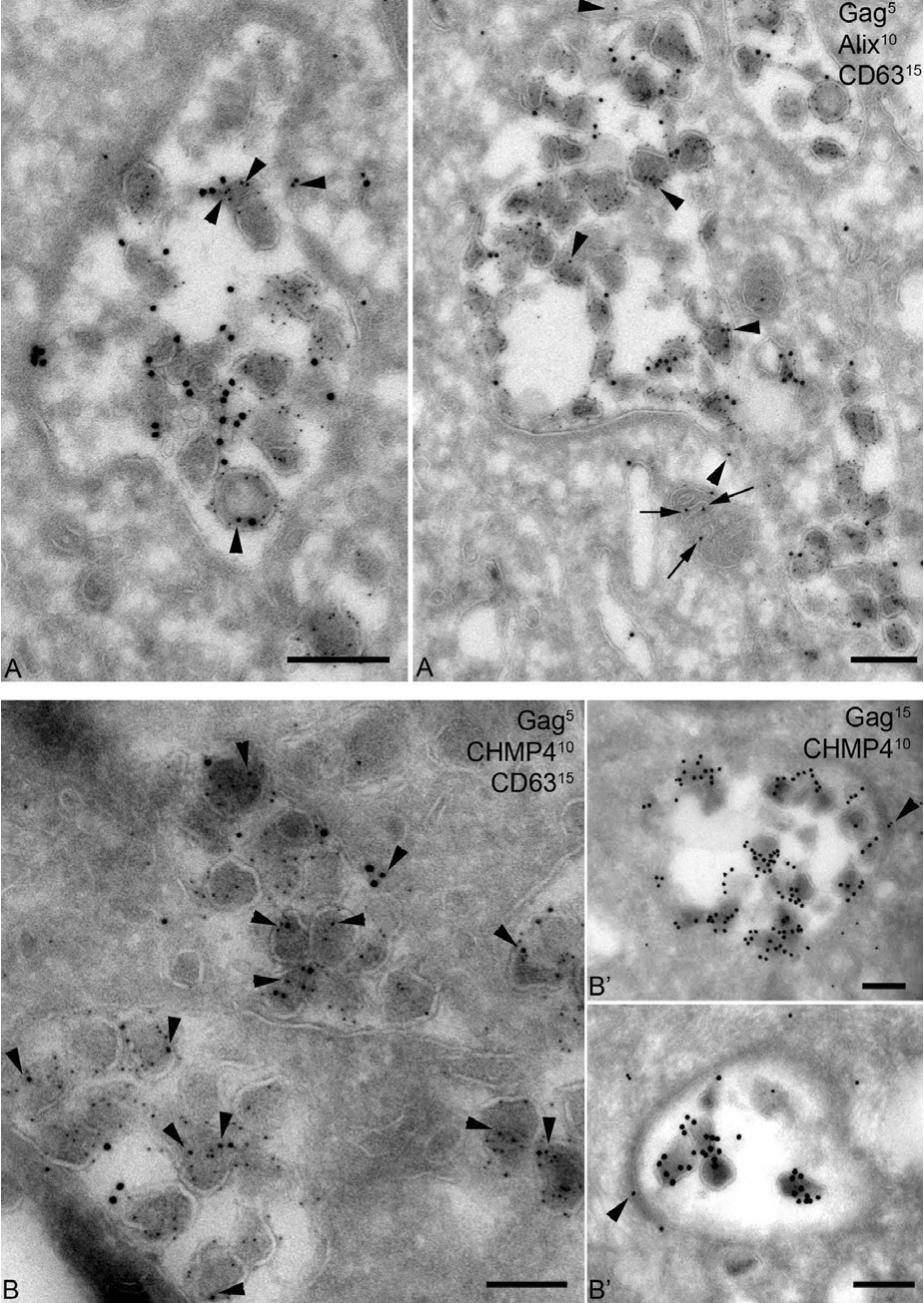
**Localization of Alix and CHMP4 at the viral assembly compartment**. Monocyte-derived-macrophages infected with HIV-1 NLAD8 for 14 days were processed for cryosectioning as described [65]. (A) Two examples of virus-containing compartments that were triple labeled for p17/p55 Gag with protein A coupled to gold particles of 5 nm or PAG5, for Alix with PAG10, and for CD63 with PAG15. Alix labeling was found on the virions and at the limiting membrane of the viral assembly compartment (black arrowheads). Note the labeled mitochondria nearby (small arrow). (B) Cryosections were triple labeled for p17/p55 Gag with PAG5, for CHMP4B with PAG10, and for CD63 with PAG15. CHMP4B was present in many virions (black arrowheads). In panels (A) and (B), CD63 was at the limiting membrane of the compartment, in small internal vesicles or incorporated in the membrane of virus particles. (B') Two examples of viral compartments double labeled for CHMP4B with PAG10, and p17/p55 Gag with PAG15. CHMP4B was associated with a thick molecular coat present at the limiting membrane of the assembly compartments (black arrowheads). Bars, 100 nm.

To our knowledge, only one team observed by Immuno-EM some ESCRT-related specific staining at the limiting membrane of these compartments [62]. However, these ESCRT-components were also present elsewhere in the cell and did not appear to be relocated to the site of viral assembly upon HIV infection [62]. In our preparations of macrophages, Alix and CHMP4 were present mainly in virions, but also at the limiting membrane of the viral assembly compartment (Figure 3). However, we did not succeed in finding other ESCRT-specific antibodies effective for immuno-EM despite testing a large collection. This difficulty may reflect the tightness of the ESCRT multi-protein complex. This also points to the limitations of the immuno-EM studies for which few antibodies can be used on ultrathin sections. Nevertheless, it is now well-accepted that the ESCRT machinery is recruited by HIV-1 in macrophages as well as in T cells at their respective locations for HIV-1 assembly, either inside the cell or at the plasma membrane.

### Nature of the viral assembly compartment in macrophages

#### Where does viral assembly take place in infected macrophages?

Initial studies suggested the existence of an intracellular compartment specialized in the assembly and storage of viral particles. Ultrastructural studies revealed budding profiles at the limiting membrane of internal compartments [63] in a process and topology similar to the biogenesis of internal vesicles or exosomes in MVBs, which are late endosomes [9]. Similar profiles were reported later [10,64,65]. Proteomic analysis of the host cell proteins incorporated into highly purified virions produced by macrophages revealed the presence of many late endosomal proteins such as MHC II, CD63, and tetraspanins [52] which is in agreement with immuno-EM studies [9,10,61]. Moreover, such virions and macrophage-derived exosomes had similar protein compositions [66]. Using recombinant viruses in which a tetracysteine tag was introduced at the C-terminus of the matrix domain of Gag, it has been possible to visualize Gag trafficking in living macrophages. Accumulations of Gag were observed both at the plasma membrane and in internal compartments carrying late endosome/MVB markers [60].

Other arguments supporting the idea that productive intracellular assembly takes place in MVB-like compartments are weak as they come from studies performed in cell lines such as HeLa, HEK 293 T, or COS cells [67-69]. Viral budding was observed in MVBs from such cells [70], while Gag was found to be transported to CD63+ MVBs in an AP3-dependent manner [44]. It has been also suggested that Gag transiently traffics through MVB-like compartment to recruit the ESCRT machinery before reaching the plasma membrane in these cell lines [71]. Recently, Joshi et al. used a HIV-1 carrying a Gag-matrix mutant (29/31KE) which localizes to MVBs in all cell types, thus showing that efficient intracellular assembly and release of viral particles occurred not only in macrophages but also in T cells [72]. This study therefore establishes that endosomal compartments can serve as productive sites for HIV-1 assembly in both T cells and macrophages.

A characteristic of the endocytic pathway is its progressive acidification which allows the activation of degradative enzymes. Endosomes would therefore constitute a hostile environment for HIV-1 which is a fragile virus sensitive to low pH and proteases [73]. However, HIV-1 remains infectious in macrophages, even after residing in macrophages for long periods of time [3]. Simultaneous identification by immuno-EM of viral assembly compartments and estimation of their pH were carried out on infected macrophages [65]. While the extended network of lysosomes present in infected macrophages was correctly acidified, viral compartments were not. Endosomal acidification is required for maturation along the endocytic pathway and fusion with lysosomes. Therefore, HIV-1 may have evolved a strategy for survival in macrophages.

It has been proposed that intracellular virions observed in HIV-1-infected macrophages represent endocytosed particles produced by neighboring cells [74]. Several arguments can be put forward to rule out this hypothesis: 1) Immuno-EM profiles obtained by several teams show viral particles at various stages of budding at the limiting membrane of the compartment [2,9,10,65]. Moreover, the viral particles seen in these compartments were often immature virions, as judged by their electron lucent material at the core and electron dense material at the periphery (see Figure 1 a schematic representation). 2) Shortly after exposure of macrophages to HIV-1, most virions are found in macropinosomes or in acidic endosomes and are subsequently degraded [65,75]. 3) In all the studies mentioned, the HIV-1 strains used expressed Vpu, which promotes virus release but also inhibits virus uptake by endocytosis [28,76]. Taken together, this strongly suggests that the majority of viral particles detected in intracellular compartments of HIV-1 infected macrophages have been de novo produced rather than recently endocytosed.

#### A compartment connected to the plasma membrane

**Figure 4:**
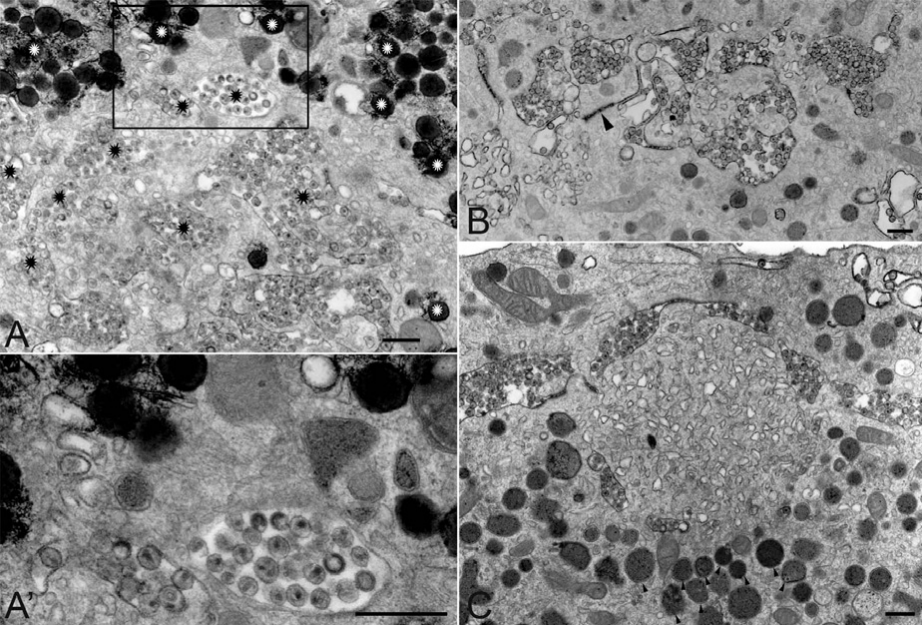
**Ruthenium red staining of HIV-1 infected macrophages**. Monocyte-derived-macrophages infected with HIV-1 (NLAD8) for 14 days were fixed on ice in the presence of ruthenium red (RR) dye and embedded in Epon for transmission electron microscopy as described [65]. (A) Viral assembly compartments negative for the RR dye were observed such as the one which is framed. Electron-dense deposits of ruthenium red-positive material were seen in lipids droplets, which lied deep within macrophages and were especially numerous near HIV-1 virus-containing vacuoles (see white asterisks). However, a majority of virus-containing compartments remained RR negative (see black asterisks). (A') Enlargement of the framed area in A. (B) Viral assembly compartments containing viral particles positive for the RR dye were also observed. Note the presence of a microchannel emanating from the central compartment (black arrowhead). (C) A "sponge-like structure" is shown in the center of the panel exhibiting highly interconnected membranes. Such structures were positive for the RR dye and very frequently were found in the vicinity of viral compartments (see above the structure). Below the structure, note the presence of numerous secondary lysosomes containing small osmiophilic particles (a few examples are pointed by black arrowheads). Bars, 400 nm.

Despite the numerous evidence showing that HIV-1 assembly occurs in macrophages in MVB-related compartments, recent studies have challenged this view. They were based on the usage of the ruthenium red (RR), which is a membrane-impermeant dye added during the fixation of infected macrophages and before their analysis by electron microscopy. Deneka et al. suggested that at least some of the virus-positive, "intracellular" structures in macrophages were actually connected to the plasma membrane via very thin microchannels allowing access of the RR dye [77]. Another team achieved similar results [64], and both concluded that the viral assembly compartment originates from the plasma membrane in infected macrophages. We also observed in our macrophage preparations that some viral compartments were RR+; however, 80% of them remained negative (Figure 4 and [65]). Interestingly, we frequently noticed in the vicinity of the viral compartments numerous electron-dense lipid droplets that were heavily stained by the RR dye (Figure 4A, see white asterisks) in agreement with the known capacity of RR to bind lipids and suggesting their connection to the extracellular space. As previously reported for other cell types [78,79], our pictures on Figure 4 reveal however the presence of electron-dense RR+ areas in the cytoplasm and mitochondria near lipid droplets, and thus indicates that the RR dye is not totally membrane-impermeant in macrophages.

A very recent study based on ion-abrasion scanning electron microscopy indicates that HIV-1-infected macrophages possess an extensive network of tubules occasionally connecting virus containing compartments with the cell surface [80]. These virion-containing tubules have a diameter of 150-200 nm and thus may differ from the narrow (< 20 nm) virion-free microchannels mentioned above. Future work will aim at confirming and quantifying the presence of these microchannels or tubules using alternative techniques.

It is currently not known whether these connections to the plasma membrane are transient or permanent. However, they may account for the lack of acidification of the viral compartment mentioned above. They could also occur as an early event during the establishment of the intracellular vacuole; or on the contrary, they may precede an exocytosis process of the viral particles, although the diameter of the microchannels appears too small to accommodate virus trafficking (around 20 nm, [77]).

Despite hundreds of EM profiles of HIV-1-infected macrophages analyzed, we never saw any budding event taking place at the plasma membrane like we observed in T cells (M. J. and P. B., unpublished observations). Importantly, three studies on macrophages showed that the viral compartments were accessible to Transferrin, but not to BSA-gold or immunoglobulin-coated gold beads added to the extracellular medium [2,64,65], supporting the concept of a compartment separated from the endocytic pathway but capable of exchanges with the recycling compartment. Alternatively, Transferrin access may be due to the microchannel connections to the plasma membrane.

Altogether it remains unclear whether the viral compartments observed in HIV-infected macrophages correspond to invaginations of the plasma membrane. We favor the notion of an intracellular compartment separated from the endocytic pathway, possessing a neutral pH and transiently connected via microchannels to the plasma membrane. However, more work is needed to resolve the nature of the viral compartment in macrophages.

#### Composition of the compartment

The limiting membrane of the compartment where viral budding takes place will eventually wrap the nascent viral particle. Therefore the lipid and protein composition of the viral membrane may reflect the origin of the assembly compartment (see [81]). The HIV-1 membrane is enriched in cholesterol, GM1 and tetraspanins, supporting the idea that HIV-1 budding could take place on lipid raft-like membranes. However, several proteins known to be normally associated with rafts like CD14 and CD45 are not found in viral envelope, whereas some proteins present in HIV-1 envelope appear excluded from lipid-rafts [66].

Tetraspanins such as CD9, CD53, CD81 and CD82 were enriched both in the compartment and in the viral membrane [10,61,82]. Although CD63 was specifically associated with HIV-1 assembly compartments in macrophages, it was dispensable for the production of infectious virus [82]. However, opposite results were obtained also in macrophages [83]. Learning more about the function of CD63, which remains elusive, will probably help to solve this discrepancy.

The limiting membrane of the viral compartment often appears to contain molecular coats (see [77] and Figure 3B') of which, the composition remains elusive. These coats are reminiscent of flat clathrin lattices found in MVBs [84] but they appear less flat, do not contain clathrin and are also observable on the microchannels connecting the compartments to the plasma membrane [77].

#### The "sponge-like structures"

Deneka et al. reported the frequent presence of "sponge-like" structures in the immediate vicinity of viral assembly compartments in infected macrophages [77]. These structures are very rich in highly interconnected membranes and accessible to the RR dye. We also observed in our macrophage preparations such RR+ structures (Figure 4C), of which the nature and function remain so far unknown. As previously noticed [77], their morphology appears similar to structures observed in primary macrophages that have been exposed to aggregated low-density lipoproteins and that are also efficiently stained by RR (see [85]).

HIV-1 is known to wrap into cholesterol-rich membranes that are required for viral production and infectivity. Since cholesterol efflux is inhibited in HIV-1-infected macrophages through a Nef-dependent mechanism [35], this accumulation of lipids may contribute to the appearance of the sponge-like structures. However, Nef does not promote the intracellular accumulation of viral particles in macrophages [3] and is dispensable for effective HIV-1 replication in macrophages [86,87]. Future work will elucidate the connection between lipid homeostasis, Nef and the assembly process in macrophages.

## Conclusions

Features of the HIV-1 cycle in macrophages still need to be better established but appear to be different at many steps from what is known during infection of CD4+ T cells (see accompanying reviews in the present issue of *Retrovirology*). Studying HIV-1 assembly in primary macrophages remains a difficult task for several reasons: Macrophages are refractory to most transfection procedures, and their very strong adherence to plastic culture dishes makes them very difficult to detach. They are terminally differentiated and thus cannot be expanded. Upon HIV infection, macrophages tend to form large syncitia and display quite a bit of donor-to-donor variability. There is a crucial need for quantitative studies that cannot be performed using conventional techniques. Several recent studies have been carried out using time-lapse based technologies, with the help of recombinant HIV-1 viruses engineered to produce fluorescent particles [20,88,89]. Recombinant viral particles can be tracked by spinning-disk confocal or TIRF microscopy. Such studies have been performed essentially with cell lines, but also in primary T cells. So far they have shed light and brought information regarding the dynamics of viral transmission between T cells, or between macrophages and T cells, and on viral entry in HeLa cells.

Despite the recent advances, many features of the HIV-1 assembly process in macrophages remain to be elucidated. Beside the exact nature and biogenesis of the viral assembly compartment, several questions have to be addressed. Among them: what are the stimuli and processes leading to the release of viral particles by infected macrophages? Is there a way of controlling this release, for example through a targeted delivery of the viral particles at the virological synapse? Given that the molecular mechanisms involved in exosome secretion are just beginning to be approached [90], a lot remains to be done. The impact of viral secretion by macrophages on cell-to-cell transmission could be very important from a physiopathological point of view, especially when highly-active anti-retroviral therapies are stopped. Virological synapses allow HIV-1 trans-infection from infected to uninfected macrophages [60]. Rapid transfer of HIV-1 particles from macrophages to autologous CD4+ T cells can occur across transient virological synapses [91]. Finally, HIV-1 also appears able to hijack tunneling nanotubes for its own spreading [92].

Another important open question is why the viral assembly compartment occurs in an internal compartment in macrophages and not in T cells. Obviously something has to differ between the two cell types, leading to distinct trafficking events. Defining the molecular basis of these phenomena may provide valuable new therapeutic targets. Among many possible hypotheses to explain the specificity of the viral assembly in macrophages, a mechanism involving the miRNA pathway could be proposed. Indeed, miRNA expression patterns are modified by HIV-1 infection [93-96], and correlate with cell permissivity to HIV-1 in the monocyte/macrophage lineage [97].

In the future, new improvements of fluorescent microscopy allowing resolution close to tens of nanometers such as photoactivated localization microscopy [98] could be used for more precise localization of Gag and other viral components. Electron tomography as well as correlative light-electron microscopy could also be of interest, especially for the fine characterization of the relation between the viral assembly compartment and the plasma membrane. No doubt that the rapid development of imaging techniques, allowing the monitoring of dynamic and rapid events with high-resolution, will benefit the field of HIV assembly in primary cells and should yield very promising and exciting findings.

## List of abbreviations

ESCRT: Endosomal Sorting Complex Required for Transport; HIV: Human Immunodeficiency Virus; Immuno-EM: immuno-electron microscopy; MHC II: Major Histocompatibility Complex class II molecules; MVBs: multi-vesicular bodies; PAG: protein A coupled to gold particles; RR: ruthenium red.

## Competing interests

The authors declare that they have no competing interests.

## Authors' contributions

PB wrote the manuscript and edited it, EB drew the figure 1 and helped to draft the manuscript, RG performed the figure 2, MS contributed to text edition, MJ performed all the EM techniques and produced the figures 3 and 4. All authors contributed to helpful discussions that enriched the review, and all authors approved the final manuscript.

## References

[B1] Gartner S Markovits P Markovitz DM Kaplan MH Gallo RC Popovic M The role of mononuclear phagocytes in HTLV-III/LAV infection Science 1986 233 215 219 10.1126/science.3014648 3014648

[B2] Orenstein JM Meltzer MS Phipps T Gendelman HE Cytoplasmic assembly and accumulation of human immunodeficiency virus types 1 and 2 in recombinant human colony-stimulating factor-1-treated human monocytes: an ultrastructural study J Virol 1988 62 2578 2586 3260631 10.1128/jvi.62.8.2578-2586.1988 PMC253687

[B3] Sharova N Swingler C Sharkey M Stevenson M Macrophages archive HIV-1 virions for dissemination in trans Embo J 2005 24 2481 2489 10.1038/sj.emboj.7600707 15920469 PMC1173148

[B4] Gonzalez-Scarano F Martin-Garcia J The neuropathogenesis of AIDS Nat Rev Immunol 2005 5 69 81 10.1038/nri1527 15630430

[B5] Ellery PJ Tippett E Chiu YL Paukovics G Cameron PU Solomon A Lewin SR Gorry PR Jaworowski A Greene WC Sonza S Crowe SM The CD16+ monocyte subset is more permissive to infection and preferentially harbors HIV-1 in vivo J Immunol 2007 178 6581 6589 17475889 10.4049/jimmunol.178.10.6581

[B6] Gordon S Taylor PR Monocyte and macrophage heterogeneity Nat Rev Immunol 2005 5 953 964 10.1038/nri1733 16322748

[B7] Steinman RM Mellman IS Muller WA Cohn ZA Endocytosis and the recycling of plasma membrane J Cell Biol 1983 96 1 27 10.1083/jcb.96.1.1 6298247 PMC2112240

[B8] Gendelman HE Orenstein JM Martin MA Ferrua C Mitra R Phipps T Wahl LA Lane HC Fauci AS Burke DS Efficient isolation and propagation of human immunodeficiency virus on recombinant colony-stimulating factor 1-treated monocytes J Ex Med 1988 167 1428 1441 10.1084/jem.167.4.1428 PMC2188914 3258626

[B9] Raposo G Moore M Innes D Leijendekker R Leigh-Brown A Benaroch P Geuze H Human Macrophages Accumulate HIV-1 Particles in MHC II Compartments Traffic 2002 3 718 729 10.1034/j.1600-0854.2002.31004.x 12230470

[B10] Pelchen-Matthews A Kramer B Marsh M Infectious HIV-1 assembles in late endosomes in primary macrophages J Cell Biol 2003 162 443 455 10.1083/jcb.200304008 12885763 PMC2172706

[B11] Gheysen D Jacobs E de Foresta F Thiriart C Francotte M Thines D De Wilde M Assembly and release of HIV-1 precursor Pr55gag virus-like particles from recombinant baculovirus-infected insect cells Cell 1989 59 103 112 10.1016/0092-8674(89)90873-8 2676191

[B12] Freed EO HIV-1 gag proteins: diverse functions in the virus life cycle Virology 1998 251 1 15 10.1006/viro.1998.9398 9813197

[B13] Bieniasz PD The cell biology of HIV-1 virion genesis Cell Host & Microbe 2009 5 550 558 10.1016/j.chom.2009.05.015 19527882 PMC3736989

[B14] Ono A HIV-1 Assembly at the Plasma Membrane: Gag Trafficking and Localization Future Virol 2009 4 241 257 10.2217/fvl.09.4 19802344 PMC2676728

[B15] Ganser-Pornillos BK Yeager M Sundquist WI The structural biology of HIV assembly Curr Opin Struct Biol 2008 18 203 217 10.1016/j.sbi.2008.02.001 18406133 PMC2819415

[B16] Klein KC Reed JC Lingappa JR Intracellular destinies: degradation, targeting, assembly, and endocytosis of HIV Gag AIDS reviews 2007 9 150 161 17982940

[B17] Lindwasser OW Resh MD Multimerization of human immunodeficiency virus type 1 Gag promotes its localization to barges, raft-like membrane microdomains J Virol 2001 75 7913 7924 10.1128/JVI.75.17.7913-7924.2001 11483736 PMC115035

[B18] Nermut MV Zhang WH Francis G Ciampor F Morikawa Y Jones IM Time Course of Gag Protein Assembly in HIV-1-Infected Cells: A Study by Immunoelectron Microscopy Virology 2003 305 219 227 10.1006/viro.2002.1692 12504555

[B19] Muriaux D Mirro J Harvin D Rein A RNA is a structural element in retrovirus particles Proc Natl Acad Sci USA 2001 98 5246 5251 10.1073/pnas.091000398 11320254 PMC33195

[B20] Hogue IB Hoppe A Ono A Quantitative FRET Microscopy Analysis of HIV-1 Gag-Gag Interaction: The Relative Contributions of CA and NC Domains, and Membrane Binding J Virol 2009 83 7322 36 10.1128/JVI.02545-08 19403686 PMC2704781

[B21] Poole E Strappe P Mok HP Hicks R Lever AM HIV-1 Gag-RNA interaction occurs at a perinuclear/centrosomal site; analysis by confocal microscopy and FRET Traffic 2005 6 741 755 10.1111/j.1600-0854.2005.00312.x 16101678

[B22] Molle D Segura-Morales C Camus G Berlioz-Torrent C Kjems J Basyuk E Bertrand E Endosomal trafficking of HIV-1 GAG and genomic RNAS regulates viral egress J Biol Chem 2009 284 19727 43 10.1074/jbc.M109.019844 19451649 PMC2740597

[B23] Jin J Sturgeon T Weisz OA Mothes W Montelaro RC HIV-1 matrix dependent membrane targeting is regulated by Gag mRNA trafficking PLoS ONE 2009 4 e6551 10.1371/journal.pone.0006551 19662089 PMC2717210

[B24] Lehmann M Milev MP Abrahamyan L Yao X-J Pante N Mouland AJ Intracellular transport of human immunodeficiency virus type 1 genomic RNA and viral production are dependent on dynein motor function and late endosome positioning J Biol Chem 2009 284 14572 14585 10.1074/jbc.M808531200 19286658 PMC2682905

[B25] McDonald B Martin-Serrano J No strings attached: the ESCRT machinery in viral budding and cytokinesis J Cell Sci 2009 122 2167 2177 10.1242/jcs.028308 19535732 PMC2723143

[B26] Van Damme N Goff D Katsura C Jorgenson RL Mitchell R Johnson MC Stephens EB Guatelli J The interferon-induced protein BST-2 restricts HIV-1 release and is downregulated from the cell surface by the viral Vpu protein Cell Host Microbe 2008 3 245 252 10.1016/j.chom.2008.03.001 18342597 PMC2474773

[B27] Van Damme N Guatelli J HIV-1 Vpu inhibits accumulation of the envelope glycoprotein within clathrin-coated, Gag-containing endosomes Cell Microbiol 2008 10 1040 1057 10.1111/j.1462-5822.2007.01101.x 18076669

[B28] Neil SJ Eastman SW Jouvenet N Bieniasz PD HIV-1 Vpu promotes release and prevents endocytosis of nascent retrovirus particles from the plasma membrane PLoS Pathog 2006 2 e39 10.1371/journal.ppat.0020039 16699598 PMC1458960

[B29] Neil SJ Zang T Bieniasz PD Tetherin inhibits retrovirus release and is antagonized by HIV-1 Vpu Nature 2008 451 425 430 10.1038/nature06553 18200009

[B30] Schindler M Rajan D Banning C Wimmer P Koppensteiner H Iwanski A Specht A Sauter D Dobner T Kirchhoff F Vpu serine 52 dependent counteraction of tetherin is required for HIV-1 replication in macrophages, but not in ex vivo human lymphoid tissue Retrovirology 2010 7 1 10.1186/1742-4690-7-1 20078884 PMC2823648

[B31] Mitchell RS Katsura C Skasko MA Fitzpatrick K Lau D Ruiz A Stephens EB Margottin-Goguet F Benarous R Guatelli JC Vpu antagonizes BST-2-mediated restriction of HIV-1 release via beta-TrCP and endo-lysosomal trafficking PLoS Pathog 2009 5 e1000450 10.1371/journal.ppat.1000450 19478868 PMC2679223

[B32] Mangeat B Gers-Huber G Lehmann M Zufferey M Luban J Piguet V HIV-1 Vpu neutralizes the antiviral factor Tetherin/BST-2 by binding it and directing its beta-TrCP2-dependent degradation PLoS Pathog 2009 5 e1000574 10.1371/journal.ppat.1000574 19730691 PMC2729927

[B33] Sato K Yamamoto SP Misawa N Yoshida T Miyazawa T Koyanagi Y Comparative study on the effect of human BST-2/Tetherin on HIV-1 release in cells of various species Retrovirology 2009 6 53 10.1186/1742-4690-6-53 19490609 PMC2702332

[B34] Adamson CS Freed EO Human immunodeficiency virus type 1 assembly, release, and maturation Adv Pharmacol 2007 55 347 387 full_text 17586320 10.1016/S1054-3589(07)55010-6

[B35] Mujawar Z Rose H Morrow MP Pushkarsky T Dubrovsky L Mukhamedova N Fu Y Dart A Orenstein JM Bobryshev YV Bukrinsky M Sviridov D Human immunodeficiency virus impairs reverse cholesterol transport from macrophages PLoS Biol 2006 4 e365 10.1371/journal.pbio.0040365 17076584 PMC1629034

[B36] Ryzhova EV Vos RM Albright AV Harrist AV Harvey T Gonzalez-Scarano F Annexin 2: a novel human immunodeficiency virus type 1 Gag binding protein involved in replication in monocyte-derived macrophages J Virol 2006 80 2694 2704 10.1128/JVI.80.6.2694-2704.2006 16501079 PMC1395445

[B37] Mayran N Parton RG Gruenberg J Annexin II regulates multivesicular endosome biogenesis in the degradation pathway of animal cells EMBO J 2003 22 3242 3253 10.1093/emboj/cdg321 12839987 PMC165635

[B38] Batonick M Favre M Boge M Spearman P Honing S Thali M Interaction of HIV-1 Gag with the clathrin-associated adaptor AP-2 Virology 2005 342 190 200 10.1016/j.virol.2005.08.001 16139856

[B39] Boge M Wyss S Bonifacino JS Thali M A membrane-proximal tyrosine-based signal mediates internalization of the HIV-1 envelope glycoprotein via interaction with the AP-2 clathrin adaptor J Biol Chem 1998 273 15773 15778 10.1074/jbc.273.25.15773 9624176

[B40] Byland R Vance PJ Hoxie JA Marsh M A conserved dileucine motif mediates clathrin and AP-2-dependent endocytosis of the HIV-1 envelope protein Mol Biol Cell 2007 18 414 425 10.1091/mbc.E06-06-0535 17108326 PMC1783771

[B41] Camus G Segura-Morales C Molle D Lopez-Verges S Begon-Pescia C Cazevieille C Schu P Bertrand E Berlioz-Torrent C Basyuk E The clathrin adaptor complex AP-1 binds HIV-1 and MLV Gag and facilitates their budding Mol Biol Cell 2007 18 3193 3203 10.1091/mbc.E06-12-1147 17538020 PMC1949356

[B42] Ohno H Aguilar RC Fournier MC Hennecke S Cosson P Bonifacino JS Interaction of endocytic signals from the HIV-1 envelope glycoprotein complex with members of the adaptor medium chain family Virology 1997 238 305 315 10.1006/viro.1997.8839 9400603

[B43] Wyss S Berlioz-Torrent C Boge M Blot G Honing S Benarous R Thali M The highly conserved C-terminal dileucine motif in the cytosolic domain of the human immunodeficiency virus type 1 envelope glycoprotein is critical for its association with the AP-1 clathrin adapter J Virol 2001 75 2982 2992 10.1128/JVI.75.6.2982-2992.2001 11222723 PMC115924

[B44] Dong X Li H Derdowski A Ding L Burnett A Chen X Peters TR Dermody TS Woodruff E Wang JJ Spearman P AP-3 directs the intracellular trafficking of HIV-1 Gag and plays a key role in particle assembly Cell 2005 120 663 674 10.1016/j.cell.2004.12.023 15766529

[B45] Joshi A Garg H Nagashima K Bonifacino JS Freed EO GGA and Arf proteins modulate retrovirus assembly and release Mol Cell 2008 30 227 238 10.1016/j.molcel.2008.03.015 18439901 PMC2386562

[B46] Lopez-Verges S Camus G Blot G Beauvoir R Benarous R Berlioz-Torrent C Tail-interacting protein TIP47 is a connector between Gag and Env and is required for Env incorporation into HIV-1 virions Proc Natl Acad Sci USA 2006 103 14947 14952 10.1073/pnas.0602941103 17003132 PMC1595456

[B47] Nishi M Ryo A Tsurutani N Ohba K Sawasaki T Morishita R Perrem K Aoki I Morikawa Y Yamamoto N Requirement for microtubule integrity in the SOCS1-mediated intracellular dynamics of HIV-1 Gag FEBS Lett 2009 583 1243 1250 10.1016/j.febslet.2009.03.041 19327355

[B48] Leblanc JJ Perez O Hope T Probing the structural states of human immunodeficiency virus type 1 pr55gag by using monoclonal antibodies J Virol 2008 82 2570 2574 10.1128/JVI.01717-07 18094163 PMC2258915

[B49] Ryo A Tsurutani N Ohba K Kimura R Komano J Nishi M Soeda H Hattori S Perrem K Yamamoto M Chiba J Mimaya J Yoshimura K Matsushita S Honda M Yoshimura A Sawasaki T Aoki I Morikawa Y Yamamoto N SOCS1 is an inducible host factor during HIV-1 infection and regulates the intracellular trafficking and stability of HIV-1 Gag Proc Natl Acad Sci USA 2008 105 294 299 10.1073/pnas.0704831105 18172216 PMC2224204

[B50] Tang Y Winkler U Freed EO Torrey TA Kim W Li H Goff SP Morse HC Cellular motor protein KIF-4 associates with retroviral Gag J Virol 1999 73 10508 10513 10559369 10.1128/jvi.73.12.10508-10513.1999 PMC113106

[B51] Martinez NW Xue X Berro RG Kreitzer G Resh MD Kinesin KIF4 regulates intracellular trafficking and stability of the human immunodeficiency virus type 1 Gag polyprotein J Virol 2008 82 9937 9950 10.1128/JVI.00819-08 18684836 PMC2566262

[B52] Chertova E Chertov O Coren LV Roser JD Trubey CM Bess JW Jr Sowder RC Barsov E Hood BL Fisher RJ Nagashima K Conrads TP Veenstra TD Lifson JD Ott DE Proteomic and biochemical analysis of purified human immunodeficiency virus type 1 produced from infected monocyte-derived macrophages J Virol 2006 80 9039 9052 10.1128/JVI.01013-06 16940516 PMC1563931

[B53] König R Zhou Y Elleder D Diamond TL Bonamy GMC Irelan JT Chiang C-Y Tu BP De Jesus PD Lilley CE Seidel S Opaluch AM Caldwell JS Weitzman MD Kuhen KL Bandyopadhyay S Ideker T Orth AP Miraglia LJ Bushman FD Young JA Chanda SK Global analysis of host-pathogen interactions that regulate early-stage HIV-1 replication Cell 2008 135 49 60 10.1016/j.cell.2008.07.032 18854154 PMC2628946

[B54] Zhou H Xu M Huang Q Gates A Zhang X Castle J Stec E Ferrer M Strulovici B Hazuda D Espeseth A Genome-Scale RNAi Screen for Host Factors Required for HIV Replication Cell Host & Microbe 2008 4 495 504 10.1016/j.chom.2008.10.004 18976975

[B55] Brass AL Dykxhoorn DM Benita Y Yan N Engelman A Xavier RJ Lieberman J Elledge SJ Identification of host proteins required for HIV infection through a functional genomic screen Science 2008 319 921 926 10.1126/science.1152725 18187620

[B56] Yeung ML Houzet L Yedavalli VSRK Jeang K-T A genome-wide short hairpin RNA screening of jurkat T-cells for human proteins contributing to productive HIV-1 replication J Biol Chem 2009 284 19463 19473 10.1074/jbc.M109.010033 19460752 PMC2740572

[B57] Bushman FD Malani N Fernandes J D'Orso I Cagney G Diamond TL Zhou H Hazuda DJ Espeseth AS König R Bandyopadhyay S Ideker T Goff SP Krogan NJ Frankel AD Young JA Chanda SK Host cell factors in HIV replication: meta-analysis of genome-wide studies PLoS Pathog 2009 5 e1000437 10.1371/journal.ppat.1000437 19478882 PMC2682202

[B58] Kok K Lei T Jin D siRNA and shRNA screens advance key understanding of host factors required for HIV-1 replication Retrovirology 2009 6 78 10.1186/1742-4690-6-78 19712452 PMC2743632

[B59] Briggs JA Johnson MC Simon MN Fuller SD Vogt VM Cryo-electron microscopy reveals conserved and divergent features of gag packing in immature particles of Rous sarcoma virus and human immunodeficiency virus Journal of Molecular Biology 2006 355 157 168 10.1016/j.jmb.2005.10.025 16289202

[B60] Gousset K Ablan SD Coren LV Ono A Soheilian F Nagashima K Ott DE Freed EO Real-time visualization of HIV-1 GAG trafficking in infected macrophages PLoS Pathog 2008 4 e1000015 10.1371/journal.ppat.1000015 18369466 PMC2267008

[B61] Kramer B Pelchen-Matthews A Deneka M Garcia E Piguet V Marsh M HIV interaction with endosomes in macrophages and dendritic cells Blood Cells Mol Dis 2005 35 136 142 10.1016/j.bcmd.2005.06.006 16087369

[B62] Welsch S Habermann A Jager S Muller B Krijnse-Locker J Krausslich HG Ultrastructural analysis of ESCRT proteins suggests a role for endosome-associated tubular-vesicular membranes in ESCRT function Traffic 2006 7 1551 1566 10.1111/j.1600-0854.2006.00489.x 17014699

[B63] Orenstein JM Ultrastructure of HIV/AIDS Ultrastruct Pathol 2002 26 245 250 10.1080/01913120290104502 12227950

[B64] Welsch S Keppler OT Habermann A Allespach I Krijnse-Locker J Krausslich HG HIV-1 Buds Predominantly at the Plasma Membrane of Primary Human Macrophages PLoS Pathog 2007 3 e36 10.1371/journal.ppat.0030036 17381240 PMC1829407

[B65] Jouve M Sol-Foulon N Watson S Schwartz O Benaroch P HIV-1 Buds and Accumulates in "Nonacidic" Endosomes of Macrophages Cell Host Microbe 2007 2 85 95 10.1016/j.chom.2007.06.011 18005723

[B66] Nguyen DG Booth A Gould SJ Hildreth JEK Evidence that HIV budding in primary macrophages occurs through the exosome release pathway J Biol Chem 2003 278 52347 52354 10.1074/jbc.M309009200 14561735

[B67] Ono A Freed EO Cell-type-dependent targeting of human immunodeficiency virus type 1 assembly to the plasma membrane and the multivesicular body J Virol 2004 78 1552 1563 10.1128/JVI.78.3.1552-1563.2004 14722309 PMC321403

[B68] Nydegger S Foti M Derdowski A Spearman P Thali M HIV-1 egress is gated through late endosomal membranes Traffic 2003 4 902 910 10.1046/j.1600-0854.2003.00145.x 14617353

[B69] Grigorov B Arcanger F Roingeard P Darlix JL Muriaux D Assembly of infectious HIV-1 in human epithelial and T-lymphoblastic cell lines J Mol Biol 2006 359 848 862 10.1016/j.jmb.2006.04.017 16682056

[B70] Sherer NM Lehmann MJ Jimenez-Soto LF Ingmundson A Horner SM Cicchetti G Allen PG Pypaert M Cunningham JM Mothes W Visualization of retroviral replication in living cells reveals budding into multivesicular bodies Traffic 2003 4 785 801 10.1034/j.1600-0854.2003.00135.x 14617360

[B71] Perlman M Resh MD Identification of an intracellular trafficking and assembly pathway for HIV-1 gag Traffic 2006 7 731 745 10.1111/j.1398-9219.2006.00428.x 16683918

[B72] Joshi A Ablan SD Soheilian F Nagashima K Freed EO Evidence that productive human immunodeficiency virus type 1 assembly can occur in an intracellular compartment J Virol 2009 83 5375 5387 10.1128/JVI.00109-09 19297499 PMC2681934

[B73] Ongradi J Ceccherini-Nelli L Pistello M Specter S Bendinelli M Acid sensitivity of cell-free and cell-associated HIV-1: clinical implications AIDS Res Hum Retroviruses 1990 6 1433 1436 10.1089/aid.1990.6.1433 2078421

[B74] Jouvenet N Neil SJ Bess C Johnson MC Virgen CA Simon SM Bieniasz PD Plasma Membrane Is the Site of Productive HIV-1 Particle Assembly PLoS Biol 2006 4 e435 10.1371/journal.pbio.0040435 17147474 PMC1750931

[B75] Marechal V Prevost M-C Petit C Perret E Heard J-M Schwartz O Human Immunodeficiency Virus Type 1 Entry into Macrophages Mediated by Macropinocytosis J Virol 2001 75 11166 11177 10.1128/JVI.75.22.11166-11177.2001 11602756 PMC114696

[B76] Harila K Prior I Sjoberg M Salminen A Hinkula J Suomalainen M Vpu and Tsg101 regulate intracellular targeting of the human immunodeficiency virus type 1 core protein precursor Pr55gag J Virol 2006 80 3765 3772 10.1128/JVI.80.8.3765-3772.2006 16571793 PMC1440481

[B77] Deneka M Pelchen-Matthews A Byland R Ruiz-Mateos E Marsh M In macrophages, HIV-1 assembles into an intracellular plasma membrane domain containing the tetraspanins CD81, CD9, and CD53 J Cell Biol 2007 177 329 341 10.1083/jcb.200609050 17438075 PMC2064140

[B78] Hayat MA Principles and Techniques of Electron Microscopy: Biological Applications 2000 4 Cambridge: Cambridge University Press

[B79] Luft JH Ruthenium red and violet. II. Fine structural localization in animal tissues Anat Rec 1971 171 369 415 10.1002/ar.1091710303 4108334

[B80] Bennett AE Narayan K Shi D Hartnell LM Gousset K He H Lowekamp BC Yoo TS Donald Bliss D EO F Subramaniam S Ion-abrasion scanning electron microscopy reveals surface-connected tubular conduits in HIV-infected macrophages PLOS Pathogens 2009 5 9 e1000591 10.1371/journal.ppat.1000591 19779568 PMC2743285

[B81] Waheed AA Freed EO Lipids and membrane microdomains in HIV-1 replication Virus Res 2009 143 162 176 10.1016/j.virusres.2009.04.007 19383519 PMC2731011

[B82] Ruiz-Mateos E Pelchen-Matthews A Deneka M Marsh M CD63 is not required for production of infectious human immunodeficiency virus type 1 in human macrophages J Virol 2008 82 4751 4761 10.1128/JVI.02320-07 18321974 PMC2346747

[B83] Chen H Dziuba N Friedrich B von Lindern J Murray JL Rojo DR Hodge TW O'Brien WA Ferguson MR A critical role for CD63 in HIV replication and infection of macrophages and cell lines Virology 2008 379 191 196 10.1016/j.virol.2008.06.029 18682304 PMC2697030

[B84] Sachse M Urbe S Oorschot V Strous GJ Klumperman J Bilayered Clathrin Coats on Endosomal Vacuoles Are Involved in Protein Sorting toward Lysosomes Mol Biol Cell 2002 13 1313 1328 10.1091/mbc.01-10-0525 11950941 PMC102271

[B85] Kruth HS Sequestration of aggregated low-density lipoproteins by macrophages Curr Opin Lipidol 2002 13 483 488 10.1097/00041433-200210000-00003 12352011

[B86] Balliet JW Kolson DL Eiger G Kim FM McGann KA Srinivasan A Collman R Distinct effects in primary macrophages and lymphocytes of the human immunodeficiency virus type 1 accessory genes vpr, vpu, and nef: mutational analysis of a primary HIV-1 isolate Virology 1994 200 623 631 10.1006/viro.1994.1225 8178448

[B87] Swingler S Mann A Jacque J Brichacek B Sasseville VG Williams K Lackner AA Janoff EN Wang R Fisher D Stevenson M HIV-1 Nef mediates lymphocyte chemotaxis and activation by infected macrophages [see comments] Nat Med 1999 5 997 103 10.1038/12433 10470075 PMC9513713

[B88] Jouvenet N Bieniasz PD Simon SM Imaging the biogenesis of individual HIV-1 virions in live cells Nature 2008 454 236 240 10.1038/nature06998 18500329 PMC2708942

[B89] Hubner W McNerney GP Chen P Dale BM Gordon RE Chuang FY Li XD Asmuth DM Huser T Chen BK Quantitative 3D video microscopy of HIV transfer across T cell virological synapses Science 2009 323 1743 1747 10.1126/science.1167525 19325119 PMC2756521

[B90] Ostrowski M Carmo NB Krumeich S Fanget I Raposo G Savina A Moita CF Schauer K Hume AN Freitas RP Goud B Benaroch P Hacohen N Fukuda M Desnos C Seabra MC Darchen F Amigorena S Moita LF Thery C Rab27a and Rab27b control different steps of the exosome secretion pathway Nature Cell Biology 2009 12 1 19 30 sup pp 1-13 10.1038/ncb2000 19966785

[B91] Groot F Welsch S Sattentau QJ Efficient HIV-1 transmission from macrophages to T cells across transient virological synapses Blood 2008 111 4660 4663 10.1182/blood-2007-12-130070 18296630

[B92] Sowinski S Jolly C Berninghausen O Purbhoo MA Chauveau A Köhler K Oddos S Eissmann P Brodsky FM Hopkins C Onfelt B Sattentau Q Davis DM Membrane nanotubes physically connect T cells over long distances presenting a novel route for HIV-1 transmission Nat Cell Biol 2008 10 211 219 10.1038/ncb1682 18193035

[B93] Yeung ML Bennasser Y Myers TG Jiang G Benkirane M Jeang KT Changes in microRNA expression profiles in HIV-1-transfected human cells Retrovirology 2005 2 81 10.1186/1742-4690-2-81 16381609 PMC1352379

[B94] Yeung ML Bennasser Y Le SY Jeang KT RNA interference and HIV-1 Adv Pharmacol 2007 55 427 438 full_text 17586323 10.1016/S1054-3589(07)55013-1

[B95] Triboulet R Mari B Lin YL Chable-Bessia C Bennasser Y Lebrigand K Cardinaud B Maurin T Barbry P Baillat V Reynes J Corbeau P Jeang KT Benkirane M Suppression of microRNA-silencing pathway by HIV-1 during virus replication Science 2007 315 1579 1582 10.1126/science.1136319 17322031

[B96] Houzet L Yeung ML de Lame V Desai D Smith SM Jeang KT MicroRNA profile changes in human immunodeficiency virus type 1 (HIV-1) seropositive individuals Retrovirology 2008 5 118 10.1186/1742-4690-5-118 19114009 PMC2644721

[B97] Wang X Ye L Hou W Zhou Y Wang YJ Metzger DS Ho WZ Cellular microRNA expression correlates with susceptibility of monocytes/macrophages to HIV-1 infection Blood 2009 113 671 674 10.1182/blood-2008-09-175000 19015395 PMC2628373

[B98] Betzig E Patterson GH Sougrat R Lindwasser OW Olenych S Bonifacino JS Davidson MW Lippincott-Schwartz J Hess HF Imaging intracellular fluorescent proteins at nanometer resolution Science 2006 313 1642 1645 10.1126/science.1127344 16902090

